# Supplementary material for: Effectiveness of Cement Augmentation on Early Postoperative Mobility in Patients Treated for Trochanteric Fractures with Cephalomedullary Nailing: A Prospective Cohort Study
Source: J Pers Med. 2022 Aug 27;12(9):1392. doi: 10.3390/jpm12091392 (PMC9501313; doi:10.3390/jpm12091392)
Supplement: Supplementary file 1 [file jpm-12-01392-s001.zip › jpm-1866738-supplementary.pdf]

Supplemental Table S1. Multivariable linear regression analysis for clinical outcomes except cumulated ambulation score

	Adjusted R^2	Adjusted beta (95% CI)	P Value
VAS during movement, day2	0.06		0.119
Age		-0.03 (-0.12 to 0.07)	0.58
Dementia		0.94 (0.16 to 1.72)	0.02
CCI		-0.22 (0.16 to 1.72)	0.31
Cement augmentation		-0.99(-2.15 to 0.44)	0.12
VAS during movement, day3	0.17		0.004
Age		-0.05 (-0.14 to 0.05)	0.31
Dementia		0.95 (0.14 to 1.76)	0.02
CCI		-0.17 (-0.62 to 0.27)	0.03
Cement augmentation		-2.36 (-3.67 to -1.06)	0.001
Barthel index, 1 week	0.44		<0.001
Age		-0.93 (-1.68 to -0.20)	0.01
Dementia		-12.59 (-18.95 to -6.24)	<0.01
CCI		-1.29 (-4.78 to 2.21)	0.46
Cement augmentation		8.60 (-1.60 to 18.79)	0.10

VAS, visual analog scale
